# Integrated Analyses of Copy Number Variations and Gene Expression in Lung Adenocarcinoma

**DOI:** 10.1371/journal.pone.0024829

**Published:** 2011-09-14

**Authors:** Tzu-Pin Lu, Liang-Chuan Lai, Mong-Hsun Tsai, Pei-Chun Chen, Chung-Ping Hsu, Jang-Ming Lee, Chuhsing Kate Hsiao, Eric Y. Chuang

**Affiliations:** 1 Graduate Institute of Biomedical Electronics and Bioinformatics, National Taiwan University, Taipei, Taiwan; 2 Graduate Institute of Physiology, National Taiwan University, Taipei, Taiwan; 3 Institute of Biotechnology, National Taiwan University, Taipei, Taiwan; 4 Department of Public Health, National Taiwan University, Taipei, Taiwan; 5 Bioinformatics and Biostatistics Core, Research Center For Medical Excellence, National Taiwan University, Taipei, Taiwan; 6 Department of Statistics and Informatics Science, Providence University, Taichung, Taiwan; 7 Division of Thoracic Surgery, Taichung Veterans General Hospital, Taichung, Taiwan; 8 Department of Surgery, National Taiwan University Hospital, Taipei, Taiwan; Medical College of Wisconsin, United States of America

## Abstract

Numerous efforts have been made to elucidate the etiology and improve the treatment of lung cancer, but the overall five-year survival rate is still only 15%. Identification of prognostic biomarkers for lung cancer using gene expression microarrays poses a major challenge in that very few overlapping genes have been reported among different studies. To address this issue, we have performed concurrent genome-wide analyses of copy number variation and gene expression to identify genes reproducibly associated with tumorigenesis and survival in non-smoking female lung adenocarcinoma. The genomic landscape of frequent copy number variable regions (CNVRs) in at least 30% of samples was revealed, and their aberration patterns were highly similar to several studies reported previously. Further statistical analysis for genes located in the CNVRs identified 475 genes differentially expressed between tumor and normal tissues (*p*<10^−5^). We demonstrated the reproducibility of these genes in another lung cancer study (*p* = 0.0034, Fisher's exact test), and showed the concordance between copy number variations and gene expression changes by elevated Pearson correlation coefficients. Pathway analysis revealed two major dysregulated functions in lung tumorigenesis: survival regulation via AKT signaling and cytoskeleton reorganization. Further validation of these enriched pathways using three independent cohorts demonstrated effective prediction of survival. In conclusion, by integrating gene expression profiles and copy number variations, we identified genes/pathways that may serve as prognostic biomarkers for lung tumorigenesis.

## Introduction

Lung cancer is the leading cause of cancer death in developed countries, and non-small cell lung carcinoma (NSCLC) accounts for the majority of lung cancers. Among NSCLCs, adenocarcinoma and squamous cell carcinoma are the two major histological types, representing 60–70% of all lung cancers. In Taiwan, most lung cancers are adenocarcinoma as well, especially among non-smoking female patients, and lung cancer mortality rates have become the highest in the world [1]. Even though numerous research efforts have been devoted to the development of lung cancer treatment over the past few decades, the overall five-year survival rate is still about 15% [2], mainly due to late diagnosis and/or lack of effective therapeutic methods. To better elucidate lung cancer etiology and identify prognostic gene sets, many studies have performed microarray analysis of gene expression profiles. While the identified gene sets indeed show significant associations with survival in their respective datasets, very few genes are common to all the different studies [3]. The discrepancy in the results of gene expression analysis may result from multiple experimental protocols, different statistical approaches, or inhomogeneous cohort characteristics. One possible strategy to increase homogeneity in these findings is to analyze gene expression in conjunction with DNA-level changes such as copy number variations (CNVs).

DNA copy number has played an important role in recent cancer studies. It explains about 12% of gene expression variations in breast cancer [4], and concordance between changes in mRNA expression levels and copy number has been observed in several genes located in copy number variable regions (CNVRs) in lung cancer [5], [6]. Furthermore, gene copy numbers have proven useful in predicting patient survival in lung cancer [7], [8]. For example, the overexpression and amplification of epidermal growth factor receptor (*EGFR*) [9], and the underexpression and loss of dual specificity phosphate 4 (*DUSP4*), correlate strongly with each other; where each serves as an effective prognostic biomarker in lung cancer [6]. Therefore, better prognostic gene sets may be identified through combined analysis of copy number and gene expression data.

Chromosome alterations, including structural changes and CNVs, have been extensively observed in tumorigenesis and are speculated to drive tumor progression in multiple cancers [10]. Accordingly, exploration of CNVs might reveal the roles they play in lung tumorigenesis. Using high resolution karyotyping techniques to scan the lung cancer genome, several aberrant regions have been detected: amplifications of 3p25–27 and 5p13–14, and deletions of 3p21 and 9q21 [11]. Further investigations of genes in these CNVRs do implicate several key players involved in lung tumorigenesis. For instance, loss of docking protein 2 (*DOK2*) as well as overexpression of baculoviral IAP repeat-containing 2/3 (*BIRC2*/*3*) can facilitate lung cancer cell proliferation and contribute to lung tumor development [12], [13]. Since genes located in these common lung cancer CNVRs are candidate oncogenes or tumor suppressors, an integrated analysis of their copy number and expression levels may provide more information about tumorigenesis in the lung.

Challenges arise, however, when integrating these multiple data sources to identify consistent and reproducible molecular signatures across different datasets. Using Venn diagrams to combine significant genes derived from different data types usually produces very few overlaps and leads to inaccurate results with high false-positive rates. The traditional single-gene approach does help to dissect complex diseases, but several limitations remain, especially the difficulties in interpretation of biological meanings when identified genes fall into non-overlapping functional categories and pathways [14]. Even when investigating cancers with similar histology, it is hard to obtain reproducibly significant gene signatures. To overcome these challenges, several studies suggested using functionally relevant gene sets instead of single-gene approaches for statistical analysis to better elucidate biological mechanisms [14], [15], [16].

In this study, we performed concurrent genome-wide microarray analyses of CNVs and gene expression in non-smoking female lung adenocarcinoma patients. By integration of these two data types, we identified 475 genes located in CNVRs that are differentially expressed between tumor and normal tissues. Pathway analysis of these dysregulated genes revealed seven significantly enriched canonical pathways, which implicated two major biological functions in lung tumorigenesis. Predictions of survival using these seven identified pathways were validated in three independent cohorts, suggesting their clinical relevance to serve as prognostic biomarkers for lung cancer.

## Results

### Frequent copy number variable regions in lung adenocarcinoma patients

**Figure 1:**
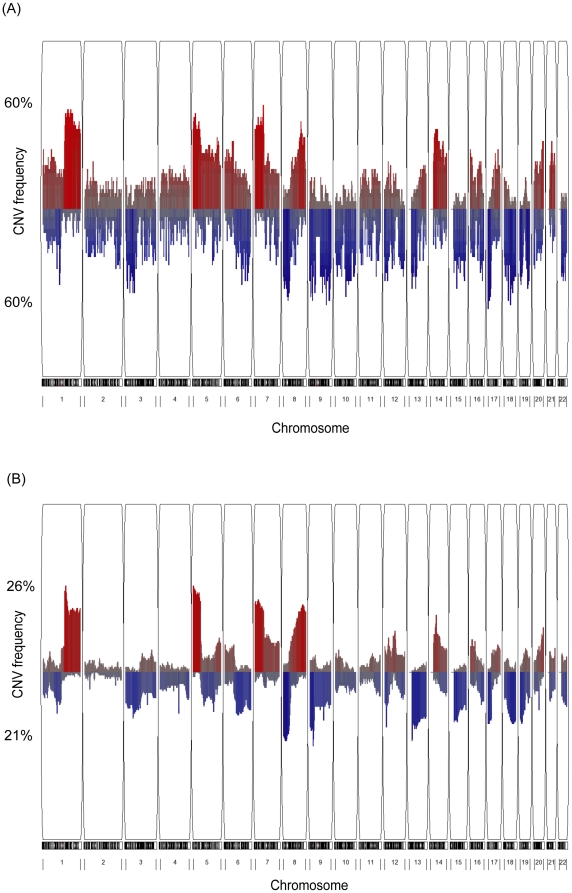
Frequency plot of CNVs in lung adenocarcinoma patients. Red color represents amplification, and blue color represents deletion. Y-axis shows the proportion of samples showing CNVs in the two datasets. (A) Our study, cohort of 42 adenocarcinoma patients. (B) The Chitale et al. study [6], cohort of 193 adenocarcinoma patients.

Copy number variation analysis was performed using Affymetrix SNP 6.0 arrays, and each tumor tissue was compared respectively to normal tissues from the same individual. As shown in Fig. 1A, several recurrent CNVRs were detected, such as the amplifications on chromosomes 1p, 5p, and 7p, and deletions on 3p, 8p, and 17p. To compare these identified CNVRs with the aberration patterns reported previously, CNV analysis was conducted on another lung adenocarcinoma cohort with both copy number and gene expression microarray data from the same individual [6]. Highly similar genomic altered patterns were observed (Fig. 1A–B), and many genes located in the CNVRs were reported as potential proto-oncogenes or tumor suppressors in lung adenocarcinoma patients [17]. For instance, amplifications of *ARNT*, *TERT*, and *NKX2-1* and deletions on *CDKN2A*, *CDKN2B* and *PIPRD* were also demonstrated in previous studies [6], [17], [18]. Among these frequent CNVRs, the most common amplification, chromosome 7p, as well as the most common deletion, chromosome 17p, occurred in approximately 60% of samples, a percentage much higher than seen in other studies [6], [17], [18]. This may imply that using adjacent normal tissue as a reference is able to reduce individual differences and to uncover more general CNVRs related to lung cancer.

### Identification of CNV-driven differentially expressed genes

**Figure 2:**
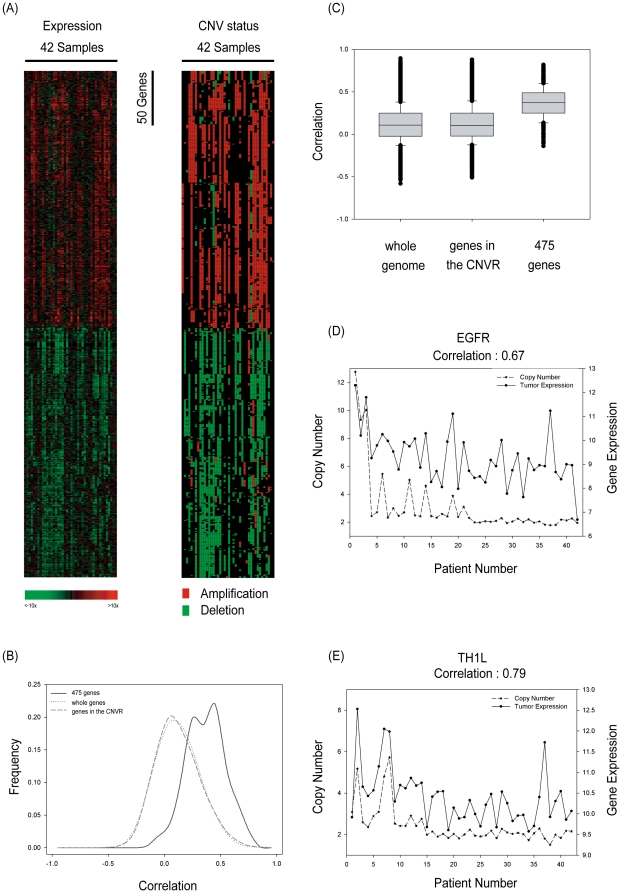
Expression profiles of CNV-driven genes. (A) Hierarchical clustering of the 475 CNV-driven genes. For gene expression (left column), the input data of each gene was normalized to its Z-value, which was obtained through two-step calculations. First, for each gene, corresponding copy number neutral samples were used as a normalization baseline, that is, the median probe intensity in the normal tissue was subtracted from probe intensities in all the samples. Next, adjusted probe intensity was divided by the standard deviation of probe intensity among copy number neutral samples to get the normalized Z-value. One-way hierarchical clustering was performed on these Z-values of gene expression. Red color indicates up-regulated genes; green color indicates down-regulated genes. For CNV status (right column), the corresponding chromosome changes are plotted in the same gene order as gene expression. Red color denotes amplification and green denotes deletion. (B) Distribution of Pearson correlation coefficients among the 475 CNV-driven genes was plotted against that from the genes located within the CNVRs. (C) Box plot of correlations among the 475 CNV-driven genes. (D–E) The Pearson correlation coefficient was utilized to describe the association between copy number and gene expression in tumor tissues for (D) *EGFR* and (E) *TH1L*. Copy number is shown on the left y-axis; gene expression is shown on the right y-axis in a log scale.

To reduce individual heterogeneities and explore the genes in the frequent CNVRs, we focused on the regions with at least 30% (13/42) of samples showing copy number changes in the following analyses. The corresponding gene expression probes within these CNVRs were mapped to 5,086 unique genes according to the annotation files provided by Affymetrix. To evaluate whether the expression levels of the 5,086 genes were associated with CNVs, patients were divided into two groups as described in the methods: the “copy number varied” group and the “copy number neutral” group. Next, for each one of such genes, an unequal variance t-test was applied to the two groups, by which we identified 609 differentially expressed genes (*p*<10^−5^, Bonferroni correction: 0.05/5,086≈10^−5^). Among them, 475 genes (78%) showing concordance in the same directional change of both CNV and gene expression were selected for further exploration. Details on these 475 genes are listed in Supplementary Table S1, and their corresponding genomic locations are shown in Supplementary Fig. S1. To validate the association between CNV status and gene expression levels of these 475 genes in tumor tissue, the gene expression data using one-way hierarchical clustering analysis was plotted in left column of Fig. 2A, and the corresponding CNV status was plotted in the right column. The heatmap revealed a highly similar co-varying pattern between gene expression and CNV (Fig. 2A). In addition to examining the dysregulated pattern among the genes, the quantitative relationships between copy number and expression level in tumor tissue were measured by using Pearson correlation coefficients (Fig. 2B). The distribution of correlation coefficients among the genes located in the CNVRs in our data showed no clear difference to that among the whole genome examined in the microarray. However, the correlation coefficients among the CNV-driven genes were substantially larger than that among other genes (Fig. 2C), suggesting that these genes were regulated by their corresponding copy numbers in lung tumor tissues. Two representative genes, *EGFR* and *TH1L*, were illustrated to demonstrate the high correlations between copy number and gene expression in tumor tissues (Fig. 2D–E). These results indicate that CNVs are important elements in driving downstream gene signaling in lung tumorigenesis.

### Comparison of identified CNV-driven genes with Chitale et al

**Figure 3:**
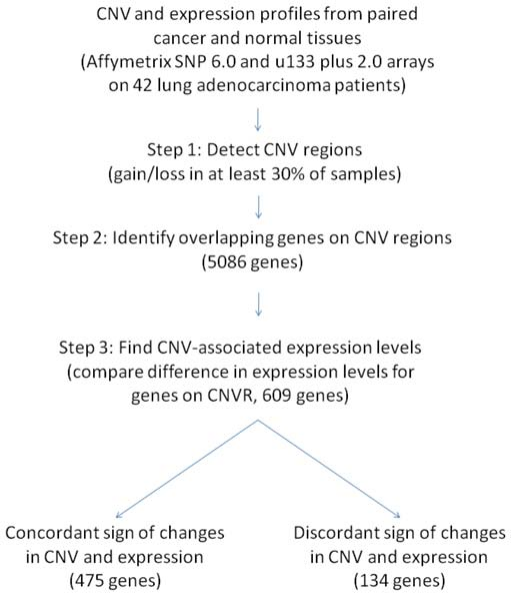
Flowchart for identifying CNV-driven genes based on CNV and expression data from paired tissues. Numbers in parentheses correspond to samples of Taiwan female lung cancer.

To further evaluate these selected 475 genes, the analysis procedures in Fig. 3 were applied to the same dataset [6] used for comparing the detected CNVRs in Fig. 1. The analysis results of Chitale et al. identified 458 differentially expressed genes (*p*<10^−4^), which were significantly overlapped with the 475 genes identified in our lung adenocarcinoma patients (*p* = 0.0034, Fisher's exact test). Next, to examine the homogeneity of these 475 genes across these two datasets, 324 genes were correspondingly mapped among the CNVRs detected in Chitale et al. Distribution and box plots of the Pearson correlation coefficients between copy number and expression level of the genes in tumor tissues demonstrated obvious elevations when compared with the total genes located in the CNVRs (Fig. S2A–B), which suggests that our proposed method efficiently identifies reproducible signatures in independent studies.

### Dysregulated biological functions and pathways of CNV-driven genes

**Figure 4:**
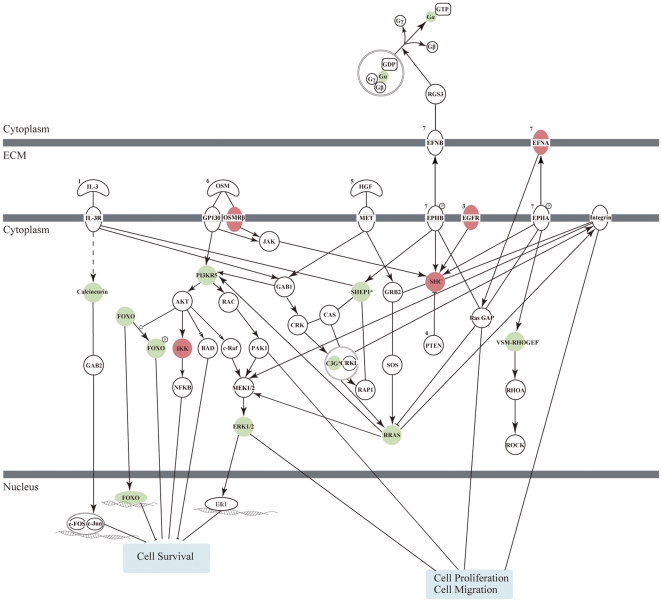
Proposed interaction network of dysregulated pathways enriched by the CNV-driven genes. Cellular response is represented by solid boxes. Genes showing amplification and up-regulation are colored in red; genes showing deletion and down-regulation are colored in green. The lines between proteins indicate evidence inferred from the literature. The superscript numbers correspond to the ranking of enrichment *p*-values.

To characterize the biological functions implicated by the 475 CNV-driven genes, Ingenuity Pathway Analysis was carried out to describe gene-gene interaction networks and canonical pathways. Fisher's exact test identified 7 canonical pathways that were significantly [−log (*p*)>2.0] enriched among the 475 CNV-driven genes (Table 1). The three pathways with the most significant *p* values included IL-3 signaling, aminoacyl-tRNA biosynthesis, and *EIF2* signaling (Table 1). IL-3 is known to trigger anti-tumor responses and retard tumor growth in NSCLC after injections [19]. A previous study reported that a tRNA synthase, hDUS2, participates in pulmonary carcinogenesis [20], though it is still not clear why genes related to aminoacyl-tRNA biosynthesis were dysregulated in lung cancer patients. *EIF2* controlled mainly protein synthesis through binding to initiator Met-tRNA^Met^ [21], and its upstream regulators were involved in the signal transduction cascade from IL-*3*. In addition to IL-*3* signaling, these genes were also downstream members shared by the other four significantly enriched pathways, and thus a proposed interaction network is displayed in Fig. 4. One major function implicated by this network was cell survival regulation via AKT signaling, which has been extensively studied and targeted in lung cancer therapy [22], [23]. In addition, there were multiple genes involved in regulating cell proliferation and cell migration through cytoskeleton reorganization, which further elucidated the biological roles these differentially expressed genes with genomic alterations may play in lung tumorigenesis.

**Table 1:** Enriched canonical pathways identified by Ingenuity Pathway Analysis among the genes with both copy number variation and differential expression.

Canonical Pathway	−log(*p*-value)^a^	Associated Gene Number^b^
**IL-3 Signaling**	2.83	7
**Aminoacyl-tRNA Biosynthesis**	2.55	5
**EIF2 Signaling**	2.37	7
**PTEN Signaling**	2.21	7
**Renal Cell Carcinoma Signaling**	2.20	6
**Oncostatin M Signaling**	2.03	4
**Ephrin Receptor Signaling**	2.02	10

a The significance level of each canonical pathway was determined by Fisher's exact test in Ingenuity Pathway Analysis.

b The associated gene number represents the number of dysregulated genes involved in the corresponding canonical pathway.

### Validation of identified pathways in three different datasets

**Figure 5:**
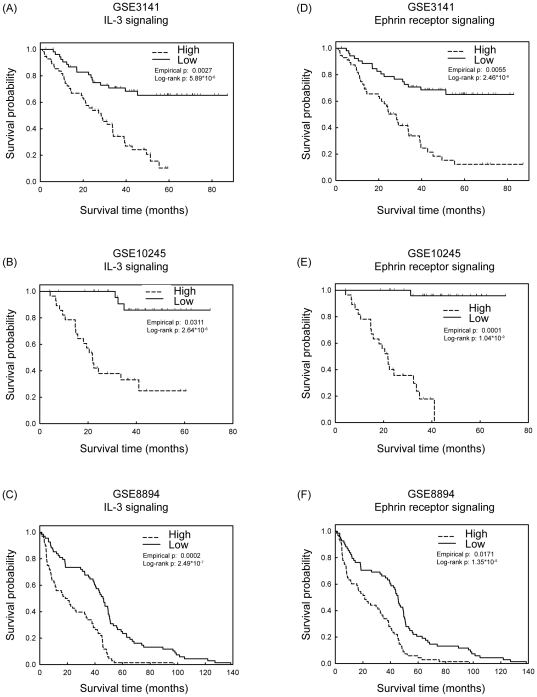
Kaplan-Meier survival curves of genes associated with IL-3 signaling or ephrin receptor signaling pathways. For each pathway in a Cox regression model, the influence of each variable was quantified by the estimated beta coefficient value. These beta values were multiplied by their original variables respectively to summarize the Cox regression score for each patient examined. Next, patients were divided into two groups according to the Cox regression scores: (1) the “High Score” group, in which scores were higher than the median scores in all samples, and (2) the “Low Score” group, in which scores were lower than the median scores in all samples. Kaplan-Meier survival analyses were performed on patients in the “High Score” and “Low Score” groups, and the empirical *p* values were determined after being compared with null baselines. (A–C) Seven genes involved in the IL-3 signaling pathway. (A) GSE3141 [24]. (B) GSE10245 [26]. (C) GSE8894 [25]. (D–F) Ten genes involved in the ephrin receptor signaling pathway. (D) GSE3141. (E) GSE10245. (F) GSE8894. Detailed gene lists were shown in Supplementary Table S4.

To validate the seven identified canonical pathways in prediction of survival probabilities, we considered three independent microarray datasets [24], [25], [26] for further investigation. (Our own dataset was unsatisfactory for validation purposes because most of the patients examined in our microarray experiments are still alive.) Detailed information about the survival evaluation procedures is described in Methods. For each dataset, the empirical *p* for testing each pathway against the null baseline is listed in Table 2. The results indicated that all genes in their respective pathways are significant survival predictors for all three datasets, except those involved in aminoacyl-tRNA biosynthesis. The two pathways with the most significant and consistent *p* values were IL-3 signaling and ephrin receptor signaling, and their corresponding survival prediction accuracy was assessed with Kaplan-Meier survival curves (Fig. 5). The prediction performances based on different numbers of genes in these two pathways were also evaluated by examining all possible combinations of the 7 or 10 genes in Kaplan-Meier survival analysis. As shown in Supplementary Fig. S3, the prediction performances improved gradually when more genes were included for survival analysis, and the lowest *p*-values were reached by using the 7 genes together (or 10 genes in the second pathway). It is worth noting that even though these CNV-driven genes were identified based on the pure lung adenocarcinoma samples, these genes demonstrated effective prediction of survival in three lung cancer datasets including patients with squamous cell carcinoma subtypes. We conclude that these differentially expressed genes with genomic alterations may not only participate in lung tumorigenesis but may also represent a prognostic signature for clinical use.

**Table 2:** Empirical *p*-values of the canonical pathways^a^.

Canonical Pathway	GSE3141	GSE10245	GSE8894
**IL-3 Signaling**	0.0027	0.0311	0.0002
**Aminoacyl-tRNA Biosynthesis**	0.1133	0.5117	0.1503
**EIF2 Signaling**	0.0182	0.0491	0.0230
**PTEN Signaling**	0.0098	0.0234	0.0048
**Renal Cell Carcinoma Signaling**	0.0018	0.0372	0.0080
**Oncostatin M Signaling**	0.0129	0.0042	0.0721
**Ephrin Receptor Signaling**	0.0055	0.0001	0.0171

a The significance levels were determined by comparison with null baselines created by random selections.

## Discussion

It is well-known that there are many causative elements contributing to cancer progression and tumorigenesis, such as transcriptional alterations, sequence mutations, and genomic changes. Among these complicated factors, CNVs have been widely reported to serve as a key driver of transcriptome dysregulation [4]. Therefore, to account for the complex relationship between copy number and gene expression, we performed an integrated analysis in paired lung adenocarcinoma tissue specimens to identify differentially expressed genes with concordant genomic alterations. Survival analyses demonstrated that the identified gene sets were consistently associated with clinical outcomes in three independent lung cancer cohorts—such consistent findings are not easily obtained by examining mRNA expression profiles alone [3].

CNV analysis provided general insights into genomic alterations in lung adenocarcinoma (Fig. 1), and the identified CNVRs were highly similar to those reported previously [6], [17], [18]. However, a notable difference was that the identified CNV frequency among our patient samples (30∼60%) was much higher, which may be attributed to the use of adjacent normal tissue, not the common reference genome, as the reference baseline. Because copy number polymorphisms exist commonly in the human genome [27], [28], comparison with the common reference genome may increase the possibility of enrolling more genomic alteration hotspots with lower degree of participation in lung tumorigenesis. Moreover, tumor tissues are usually inhomogeneous across patients, and thus incorporating adjacent normal tissue from the same individual into the analysis may help to reduce variations caused by individual heterogeneity.

To identify differentially expressed genes involved in lung tumorigenesis, we applied statistical analyses on gene expression data between tumor and normal tissues rather than between tumor tissues with and without copy number variations. Similar patterns of fold changes were illustrated in both analyses (Fig. S4), but minor differences were observed while examining tumor tissues only. It is possible that gene expression profiles had already been dysregulated to adapt to proper function in tumor tissues during carcinogenesis. Therefore, even though adjacent normal tissues may be partly contaminated by tumor samples, they still provide a better comparison baseline, which resembled regular gene expression profiles in healthy lung.

Compared to other cytogenetic reports about lung cancer, we observed different genomic states on chromosome 5q. Several studies reported the association between deletion of this chromosomal region and small cell and squamous cell lung cancer subtypes [29], [30], [31], and it was also pointed out that this deletion may be associated with smoking history [32]. Nevertheless, amplifications of 5q have been detected in other adenocarcinoma studies [33], [34]. This controversy may come from differences in lung cancer subtypes and/or in history of smoking. Here, in our study, only women with non-smoking lung adenocarcinoma were recruited and thus no comparison group is available. For further investigation to elucidate the role this region may play, data from smoking females may be of help.

Regarding the 5,086 genes residing in the CNVRs, significantly different expression associated with CNVs was detected in 609 genes (12%), a proportion comparable to that of previous studies [4], [35]. Among the 609 selected genes, 475 genes (78%) showed positive correlation between CNV and mRNA expression and 134 genes (22%) showed negative correlation. The most positively correlated gene, *C20orf11*, was identified here but no functional study is available at this time. However, the second gene, *TH1L*, has been shown to play an important role in many processes of inflammation and pulmonary fibrosis in lung [36], and there were two other reports indicating that *TH1L* may be associated with tumor development [37], [38]. The knockdown of *TH1L* was able to trigger several molecular and cellular changes correlated with epithelial-mesenchymal–transitition in MCF7 cells [37], and *TH1L* participates in the regulation of MAPK signaling [38], which was closely associated with lung cancer. In addition, the elevated correlations of the 475 concordantly changed genes further evidenced that our statistical approaches are able to efficiently identify dysregulated genes based on CNVs.

On the other hand, the reason why the other 134 genes displayed discordant changes remains unclear. The occurrence of negative correlation could result from just random chance, or, alternatively, from the existence of other regulatory mechanisms that inhibit genomic alterations, such as miRNA regulations, gene mutations, and epigenetic methylations [39]. Tumor tissues may suffer damage if essential genes for tumor development undergo CNVs that amplify tumor suppressors or delete oncogenes. For instance, both the most negatively correlated gene, *RTN1*, involved in detoxification in lung cancer [40], and a potential lung cancer tumor suppressor, *SEMA5A* [41], were significantly down-regulated though frequently amplified in tumor tissues. Therefore, the relevance of these discordant genes to lung cancer deserves further investigation.

To further explore whether these 475 CNV-driven genes were sample dependent, the same statistical approaches shown in Fig. 3 were performed only in non-smoking lung cancer women from the Chitale et al. study [6]. After excluding those without both CNV and gene expression data, only 28 samples were remained for further analysis. The results showed higher similarity in the gene list (80% vs. 68%), and lower significant levels of overlapping with our data (p = 0.000005 versus p = 0.0034, Fisher's exact test). Moreover, the CNV frequency of amplifications at 5q was much higher (∼25%) in these non-smoking female patients (Fig. S5), which agreed with previous report that smoking history was associated with the deletion of 5q [32]. Since similar results were observed in female non-smokers and mixed population, these results indicated that our algorithm in integrating copy number variation with gene expression could be applied to other types of lung cancer.

The major cellular function implicated by the interaction network summarized from the 7 canonical pathways was cell survival regulation (Fig. 4). It is well-known that *EGFR* participates in the development and progression of lung cancer [42], and its amplifications and mutations correlate with effective response to several *EGFR* tyrosine kinase inhibitors (TKIs) for NSCLC therapy [43]. Better treatment outcomes of *EGFR*-TKIs were shown in females, non-smokers, and patients with lung adenocarcinoma, and thus it is not surprising to observe frequent amplifications (52%) and mutations (83%) of *EGFR* in our results. *IKBKE* was indicated as a potential oncogene by phosphorylating inhibitors of *NFKB* to prolong cell survival, and its amplifications and over-expressions were seen in over 30% of breast cancer patients and cell lines [44]. Though the deletions of *ERK2* seemed to be contradictory, recurrent loss of 22q (29%) was also detected in another study with a similar population [33]. Minor expression ratio changes between tumor and normal tissues were observed in this study (0.7∼1.1) and the other three lung cancer cohorts (0.9∼1.1) examining paired samples [45], [46], [47]. Moreover, activation of *ERK2* signaling requires phosphorylation [48], which is beyond the detection scope of gene expression microarrays, and thus ongoing research efforts are warranted to further elucidate such mechanisms. In addition to cell survival, cell migration through regulation of integrin was another function implicated by this interaction network. Integrin-dependent interaction with the surrounding extracellular matrix correlates with invasive abilities in lung cancer and other cancer types [49]. Lastly, the proposed interaction network was similar to one identified by integrating CNVs and sequence alterations in both breast and colorectal cancers [50], which suggests that these dysregulated genes are highly associated not only with lung tumorigenesis, but also with multiple cancers.

The survival predictions using these CNV-driven genes were effective in lung adenocarcinoma and, surprisingly, in squamous cell carcinoma patients as well (Table 2). To further explore whether these CNV-driven genes are independent of lung cancer subtype, the survival prediction was examined in NSCLC patients with only squamous cell carcinoma [51]. Although less satisfactory results were observed (Table S2), several genes involved in the ephrin receptor signaling pathway still showed effective prediction ability, concurring that genes participation in regulation of integrin may become dysregulated during the tumorigenic process across different cancer types [49]. Therefore, these dysregulated genes with CNVs may become promising targets for further pharmacological research in cancer therapy.

## Materials and Methods

### Ethics Statement

Written informed consent was obtained from all subjects and/or guardians for the use of their tissue samples. This study was approved by National Taiwan University Hospital Research Ethics Committee and The Institutional Review Board of Taichung Veterans General Hospital.

### Sample preparation and microarray experiments

One hundred and twenty paired lung tumor and adjacent normal tissues were collected from patients admitted to National Taiwan University Hospital or Taichung Veterans General Hospital. Fourty-two pairs of lung tumor and normal specimens from non-smoking adenocarcinoma female patients were analyzed by using Affymetrix SNP 6.0 and Affymetrix U133plus2.0 microarrays, after extraction of DNA and RNA according to the manufacturers' instructions. The mean ± SD age of these samples was 62±10 years, and 71% (30/42) of the patients were in stage I or II. Summary patient characterisitics are shown in Table S3. The microarray data have been submitted to the Gene Expression Omnibus database (accession number GSE19804).

### Identification of CNV-driven differentially expressed genes

To investigate genomic alternations, we used an Affymetrix Genome-Wide Human SNP 6.0 array containing 1.8 million SNP and CNV probes in total. The microarray data were imported into the Partek Genomic Suite to perform CNV analysis. Since both tumor and normal tissues from the same individual were examined, each tumor tissue could be compared with its counterpart, the normal tissue, respectively. A genomic segment was defined if the following criteria were all satisfied: minimum consecutive genomic markers ≥100, *p*-value≤0.001, and signal-to-noise ratio (SNR)≥0.3. These identified segments were indicated as copy number variated if their copy number changes were at least 0.3; that is the copy number of an amplified region was higher than 2.3, and the copy number of a deleted region was lower than 1.7, respectively. Next, to identify CNVs common to all lung adenocarcinomas, only regions showing changes in at least 30% (13/42) of the samples were analyzed further (step 1 in Fig. 3). The overlapping genes within these identified CNVRs were obtained after searching through the Affymetrix annotation file version 30 (step 2: 5,086 genes). To evaluate whether expression of these genes was related to CNV, patients were classified into two groups according to their CNV status: one group is for copy number variated (gain/loss), and the other group for copy number neutral. For each one of the 5,086 genes, an unequal variance *t*-test was applied to the gene expression variation between copy number variated tumor tissues and copy number neutral normal tissues (step 3: 609 genes). To identify CNV-driven genes, only genes with concordant changes in copy number and gene expression were collected for further analyses (step 4: 475 genes). Visualization of the identified genes, including CNV statuses and expression levels in tumor tissues, was illustrated by hierarchical clustering in the Genesis program [52] in Fig. 2.

### Comparison of identified CNV-driven genes with Chitale et al

To further demonstrate the usefulness of the procedures in the flowchart for identifying CNV-driven genes (Fig. 3), another lung cancer dataset with copy number and gene expression data from the same individual was investigated [6]. The same analysis procedures were applied, except that the comparision of differentially expressed genes in step 3 was conducted on only tumor tissues. In other words, gene expression variables were examined on tumor tissues between copy number variated and neutral samples since no adjacent normal tissues were studied in this cohort.

### Validation of CNV-driven genes and pathways with three different datasets

**Figure 6:**
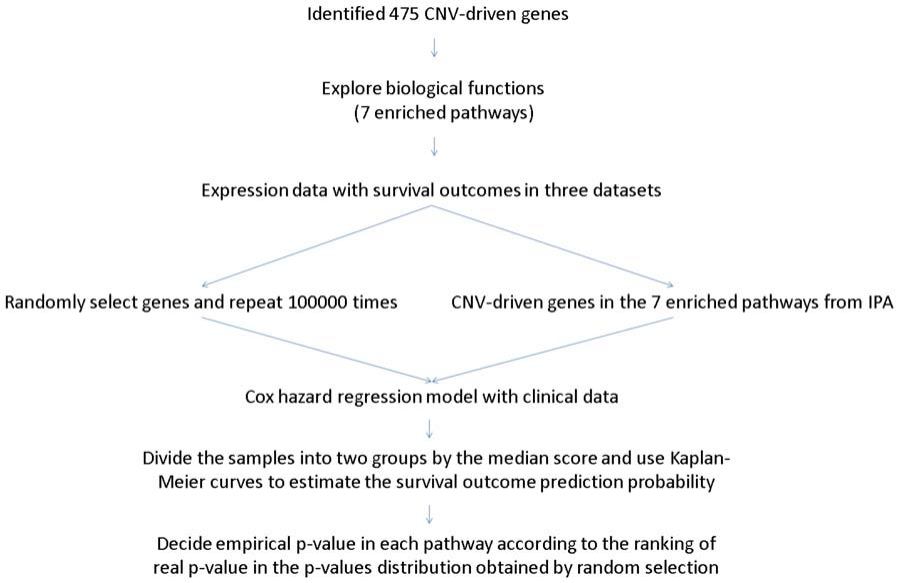
Flowchart for clinical validation of CNV-driven genes. Three independent lung cancer datasets retrieved from Gene Expression Omnibus [53] were examined: GSE3141 [24], GSE8894 [25], and GSE10245 [26].

To characterize which biological functions and canonical pathways the significantly differentially expressed genes are part of, Ingenuity Pathway Analysis was carried out. After identifying the pathways enriched by the CNV-driven genes, their performance in prediction of survival probabilities was evaluated. Here, we considered three microarray datasets (summary statistics are given in Table 3) with published survival outcomes [24], [25], [26], which were retrieved from Gene Expression Omnibus [53], to conduct the following validation procedures (Fig. 6). Since intensity distributions were usually inconsistent in different studies, the probe intensities were first standardized across all the patients respectively by the Z-score method. For the genes involved in a specifc canonical pathway, the Cox regression model was used to evaluate the association between expression of these CNV-driven genes and survival outcomes with the available clinical data. In a Cox regression model, the influence of each variable was quantified by the estimated beta coefficient value. These beta values were multiplied by their original variables respectively to summarize the Cox regression score for each patient examined. Next, patients were divided into two groups according to the Cox regression scores: (1) the “High Score” group, in which scores were higher than the median scores in all samples, and (2) the “Low Score” group, in which scores were lower than the median scores in all samples. Kaplan-Meier survival analyses were performed on patients in the “High Score” and “Low Score” groups to evaluate the association between CNV-driven genes and survival outcomes.

**Table 3:** Sample characteristics of the three microarray datasets used for survival evaluation.

Characteristics	GSE3141	GSE10245	GSE8894^a^^,^ b
**Sample number**	111	58	136
**Microarray platform**	Affymetrix U133plus2.0	Affymetrix U133plus2.0	Affymetrix U133plus2.0
**Age**	NA	64.5±9.3	60.8±9.6
**Tumor types**			
Adenocarcinoma	58 (52%)	40 (69%)	60 (44%)
Squamous	53 (48%)	18 (31%)	76 (56%)
**Gender**	NA		
Male		44 (76%)	103 (24%)
Female		14 (24%)	33 (76%)

a Two samples were removed due to lack of age information.

b Recurrence-free survival was used here.

Furthermore, to establish a null baseline for comparison, Cox regression and Kaplan-Meier survival analyses were conducted again but with genes randomly selected from the original pool, where the number of genes was kept the same as the pathway under study. To incorporate the heterogeneity of the genes selected, 100,000 iterations were performed for each pathway. Empirical *p* values of the identified pathways were then determined by comparing the Kaplan-Meier survival prediction probability with the null baselines; that is, the ranking of the Kaplan-Meier *p*-values.

## Supporting Information

Figure S1 **Genomic locations of the CNV-driven genes.** Y-axis shows the proportion of samples showing CNVs.
(TIF)

Figure S2 **Pearson correlation coefficients of the 324 overlapped CNV-driven genes in the Chitale et al. study.** (A) Distribution of correlations among the CNV-driven genes was plotted against that from the genes located within the CNVRs. (B) Box plot of correlations among the 324 CNV-driven genes.
(TIF)

Figure S3 **Prediction performances based on different numbers of genes in the IL-3 signaling and ephrin receptor signaling pathways.** Kaplan-Meier survival curves were used to evaluate the prediction performances using all possible combinations of the 7 or 10 genes within the two pathways. X axis denotes the number of genes used in survival analysis, and Y axis represents the corresponding average Kaplan-Meier −log *p*-values.
(TIF)

Figure S4 **Fold changes of expression relative to normal or tumor tissues.** Relative expression level is shown on the x-axis in a log scale; frequency of genes is shown in the y-axis.
(TIF)

Figure S5 **Frequency plot of CNVs in the non-smoking lung adenocarcinoma women from the Chitale et al. study.** Red color represents amplification, and blue color represents deletion. Y-axis shows the proportion of samples showing CNVs in the dataset.
(TIF)

Table S1 **Statistics of the 475 CNV-driven genes.**
(PDF)

Table S2 **Empirical** ***p*** **-values of the canonical pathways in GSE4573^a^**
(PDF)

Table S3 **Sample characteristics of lung cancer patients examined by both Affymetrix SNP6.0 and Affymetrix U133plus 2.0 arrays.**
(PDF)

Table S4 **Genes involved in the IL-3 signaling and ephrin receptor signaling pathways.**
(PDF)
