# Supplementary figures and images for: Integrated Analyses of Copy Number Variations and Gene Expression in Lung Adenocarcinoma
Source: PLoS One. 2011 Sep 14;6(9):e24829. doi: 10.1371/journal.pone.0024829 (PMC3173487; doi:10.1371/journal.pone.0024829)

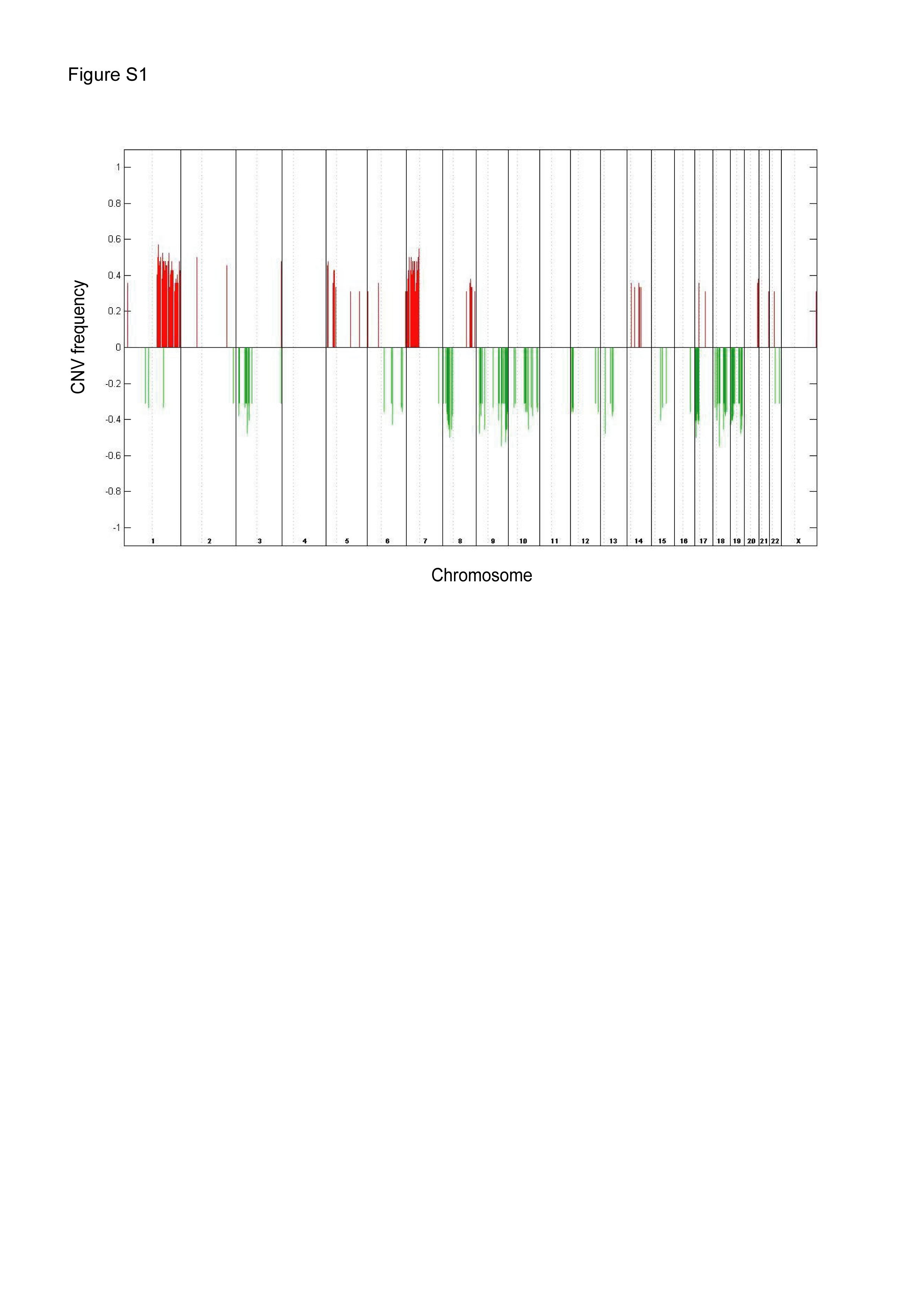

Supplement: Figure S1 — Genomic locations of the CNV-driven genes. Y-axis shows the proportion of samples showing CNVs. (TIF) [file pone.0024829.s001.tif]

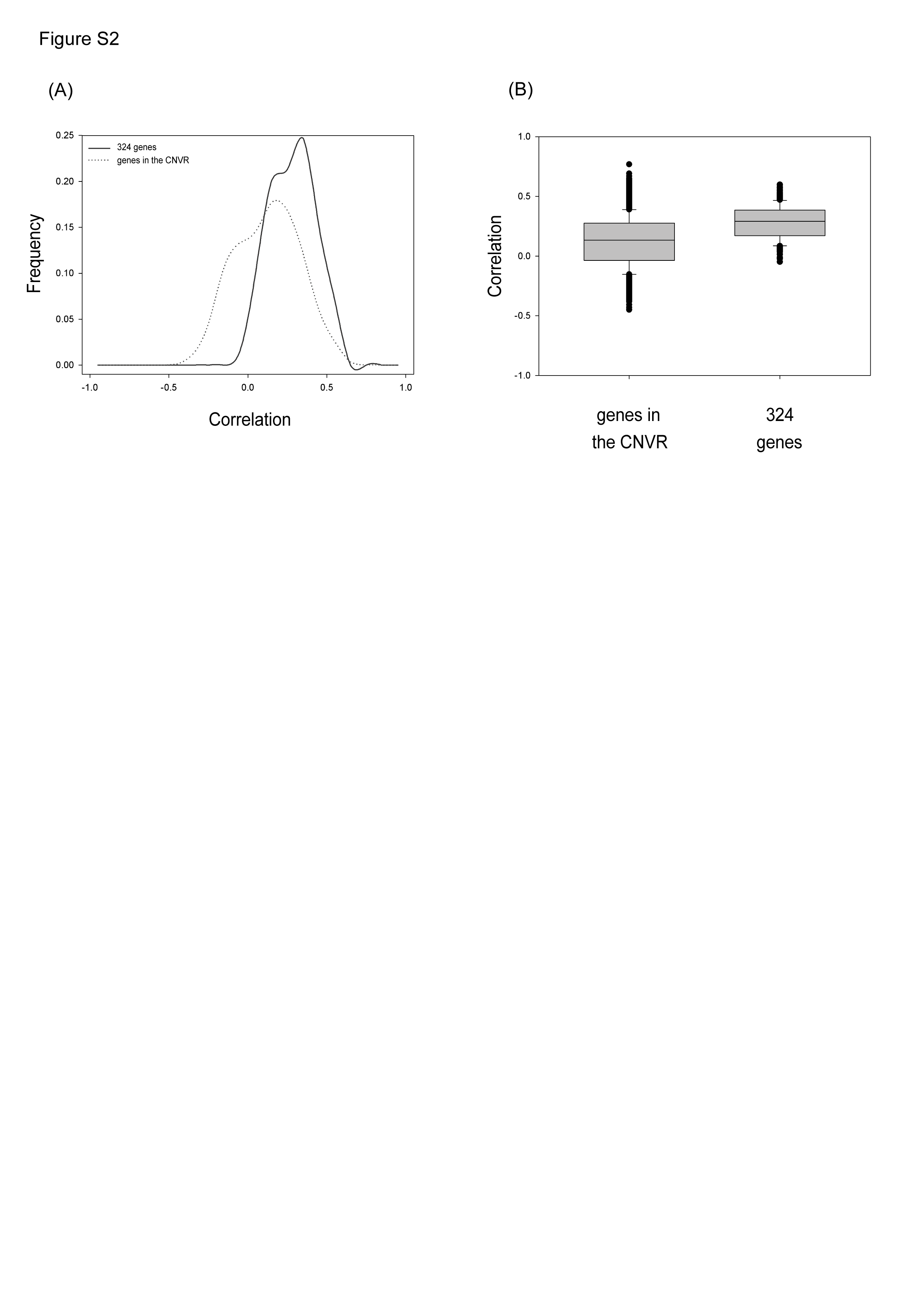

Supplement: Figure S2 — Pearson correlation coefficients of the 324 overlapped CNV-driven genes in the Chitale et al. study. (A) Distribution of correlations among the CNV-driven genes was plotted against that from the genes located within the CNVRs. (B) Box plot of correlations among the 324 CNV-driven genes. (TIF) [file pone.0024829.s002.tif]

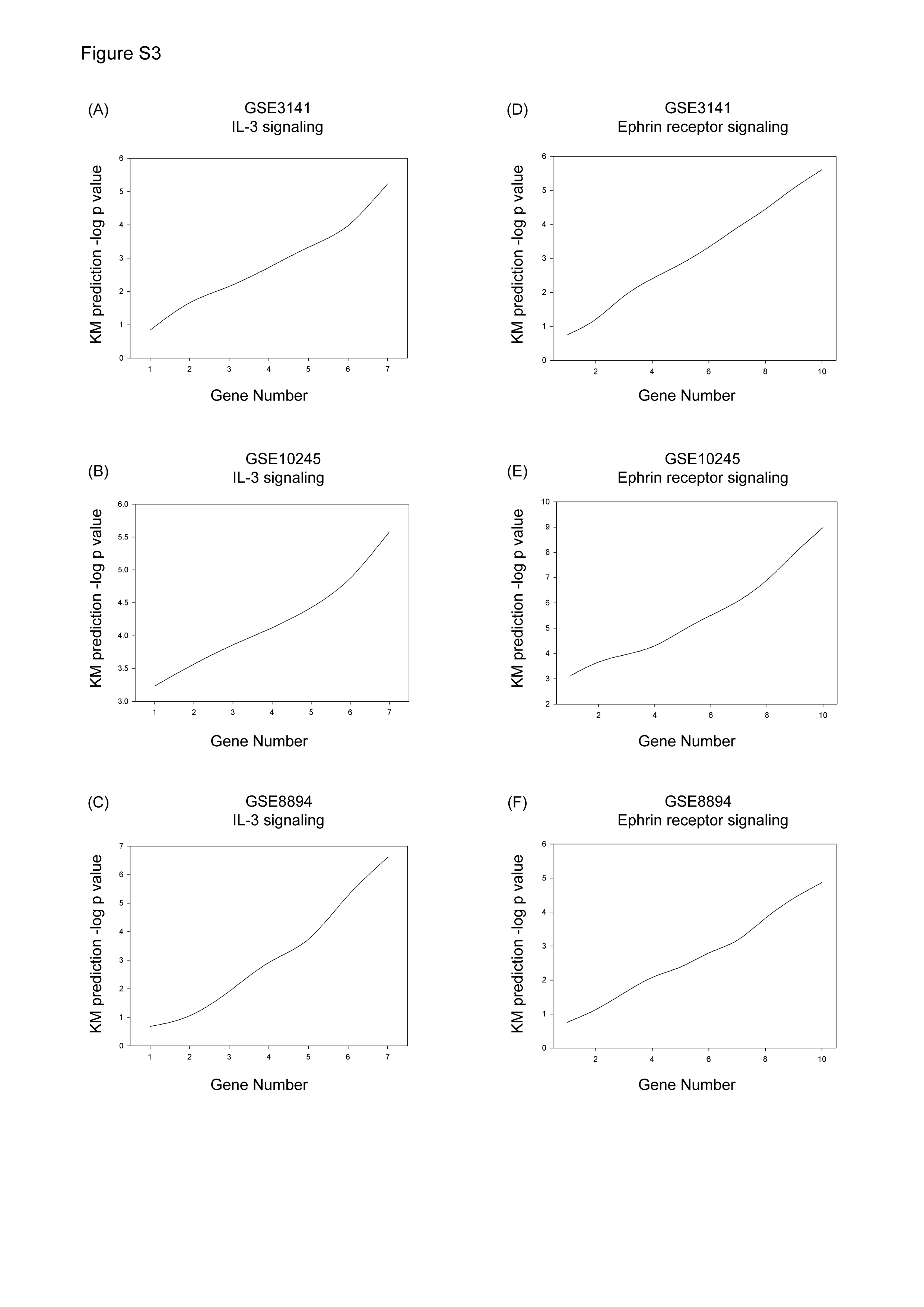

Supplement: Figure S3 — Prediction performances based on different numbers of genes in the IL-3 signaling and ephrin receptor signaling pathways. Kaplan-Meier survival curves were used to evaluate the prediction performances using all possible combinations of the 7 or 10 genes within the two pathways. X axis denotes the number of genes used in survival analysis, and Y axis represents the corresponding average Kaplan-Meier −log p-values. (TIF) [file pone.0024829.s003.tif]

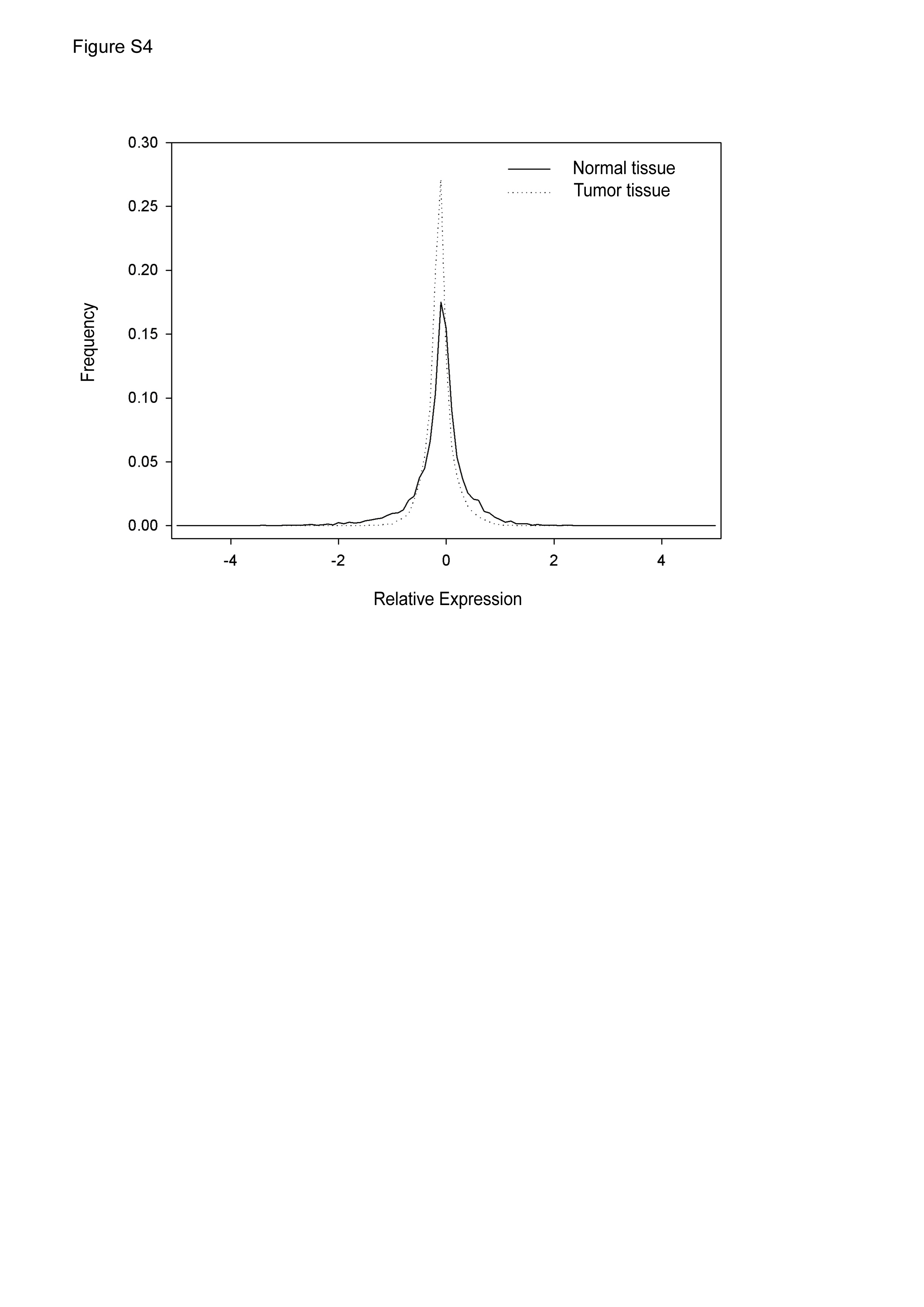

Supplement: Figure S4 — Fold changes of expression relative to normal or tumor tissues. Relative expression level is shown on the x-axis in a log scale; frequency of genes is shown in the y-axis. (TIF) [file pone.0024829.s004.tif]

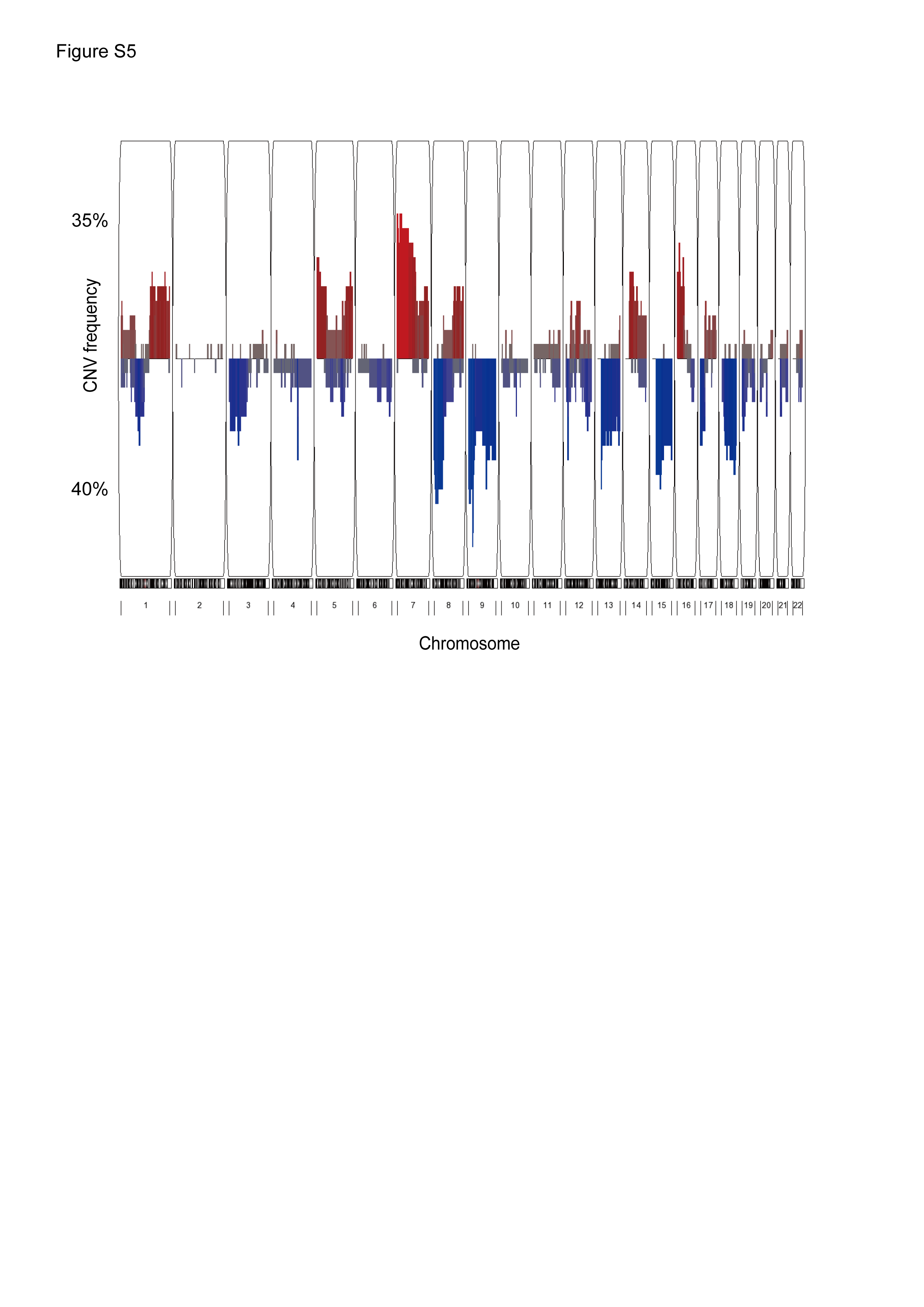

Supplement: Figure S5 — Frequency plot of CNVs in the non-smoking lung adenocarcinoma women from the Chitale et al. study. Red color represents amplification, and blue color represents deletion. Y-axis shows the proportion of samples showing CNVs in the dataset. (TIF) [file pone.0024829.s005.tif]
