# Supplementary material for: Integrated Analyses of Copy Number Variations and Gene Expression in Lung Adenocarcinoma
Source: PLoS One. 2011 Sep 14;6(9):e24829. doi: 10.1371/journal.pone.0024829 (PMC3173487; doi:10.1371/journal.pone.0024829)
Supplement: Table S1 — Statistics of the 475 CNV-driven genes. (PDF) [file pone.0024829.s006.pdf]

Gene Symbol	CNV Status	CNV Frequency	*p*-value	Log2 Expression Ratio
ACBD6	Amplification	47.62	4.63E-08	0.66
ACP6	Amplification	50	5.67E-08	0.94
ADAR	Amplification	45.24	5.05E-08	0.68
AGR2	Amplification	42.86	6.66E-10	1.79
AGT	Amplification	33.33	1.73E-09	2.26
AMACR	Amplification	42.86	5.31E-10	1.38
ANLN	Amplification	42.86	6.06E-10	3.12
ANXA9	Amplification	33.33	3.31E-06	0.83
APOA1BP	Amplification	35.71	4.14E-09	1.06
ASPM	Amplification	52.38	1.85E-06	2.11
AVL9	Amplification	30.95	5.19E-19	1.78
B4GALT3	Amplification	30.95	3.71E-07	0.75
BCL9	Amplification	47.62	2.71E-07	0.76
BOLA1	Amplification	42.86	2.39E-06	0.74
BZW2	Amplification	42.86	3.52E-12	1.3
C14orf101	Amplification	33.33	1.88E-06	0.54
C14orf166	Amplification	35.71	1.13E-09	0.65
C16orf13	Amplification	35.71	1.80E-09	0.81
C1GALT1	Amplification	38.1	7.58E-07	1.33
C1orf106	Amplification	33.33	1.59E-14	2.37
C1orf107	Amplification	47.62	4.45E-09	0.87
C1orf112	Amplification	50	5.35E-09	1.47
C1orf27	Amplification	45.24	4.83E-07	0.95
C1orf31	Amplification	40.48	6.88E-09	0.84
C1orf35	Amplification	35.71	5.67E-09	0.54
C1orf53	Amplification	52.38	6.47E-08	2.13
C1orf74	Amplification	47.62	1.88E-09	0.63
C20orf11	Amplification	40.48	1.31E-09	1.12
C20orf20	Amplification	40.48	5.01E-08	1.15
C5orf22	Amplification	33.33	9.91E-09	0.69
C5orf34	Amplification	33.33	1.74E-07	0.67
C7orf10	Amplification	30.95	1.07E-07	1.28
C7orf20	Amplification	33.33	6.02E-10	1
C7orf30	Amplification	30.95	3.82E-07	0.75
C7orf44	Amplification	35.71	4.86E-08	1.04
C7orf46	Amplification	40.48	4.00E-06	0.92
C7orf50	Amplification	30.95	6.18E-08	0.89
CABLES2	Amplification	38.1	5.56E-07	1.32
CACYBP	Amplification	47.62	1.53E-08	0.74
CBX3	Amplification	30.95	9.36E-12	0.9
CCDC127	Amplification	47.62	9.57E-07	0.58
CCL28	Amplification	30.95	1.22E-06	1.02
CCM2	Amplification	30.95	1.40E-06	0.63
CCT5	Amplification	47.62	2.41E-08	0.75
CCT6A	Amplification	35.71	1.96E-11	1.33

CD1A	Amplification	40.48	6.83E-06	1.24
CD2AP	Amplification	30.95	3.26E-06	0.93
CDCA7L	Amplification	40.48	3.54E-06	1.07
CENPF	Amplification	42.86	5.41E-09	2.25
CENPL	Amplification	38.1	5.20E-06	0.98
CHCHD2	Amplification	35.71	2.01E-13	1
CHD1L	Amplification	47.62	7.53E-11	0.97
CKS1B	Amplification	33.33	4.20E-09	1.28
CLPTM1L	Amplification	47.62	2.11E-09	1.13
COG2	Amplification	35.71	4.83E-11	0.75
COPA	Amplification	40.48	6.06E-08	0.58
CRABP2	Amplification	40.48	2.41E-08	2.55
DAP	Amplification	47.62	8.28E-09	1.09
DARS2	Amplification	38.1	2.27E-07	0.7
DCAF13	Amplification	30.95	2.39E-06	0.97
DDX56	Amplification	30.95	1.64E-07	0.96
DNAH14	Amplification	33.33	3.28E-11	1.57
DPY19L1	Amplification	47.62	1.13E-08	1.3
DUSP23	Amplification	50	3.99E-06	0.91
EEF1A2	Amplification	38.1	4.77E-06	1.37
EFNA3	Amplification	33.33	5.40E-07	0.52
EFNA4	Amplification	33.33	5.00E-14	1.95
EFNA5	Amplification	30.95	5.35E-06	1.14
EGFR	Amplification	52.38	3.10E-06	1.42
EGLN3	Amplification	33.33	3.97E-07	1.28
EIF3H	Amplification	30.95	9.81E-06	0.39
ENAH	Amplification	30.95	1.17E-06	0.98
EPRS	Amplification	30.95	3.23E-11	0.8
ETNK2	Amplification	38.1	4.06E-07	0.84
EXO1	Amplification	35.71	2.48E-08	1.3
FAM173A	Amplification	33.33	1.15E-07	0.8
FAM173B	Amplification	47.62	3.39E-06	0.73
FBXO32	Amplification	33.33	2.27E-11	1.41
FCRL5	Amplification	47.62	8.36E-07	1.64
FH	Amplification	47.62	3.49E-09	0.78
FIGNL1	Amplification	50	1.10E-08	1.87
FLAD1	Amplification	33.33	2.08E-09	0.88
FLVCR1	Amplification	33.33	9.08E-09	1.45
GALNT2	Amplification	33.33	5.68E-06	0.54
GARS	Amplification	40.48	7.15E-06	0.73
GBAS	Amplification	35.71	1.75E-07	1.07
GGCT	Amplification	40.48	2.40E-12	1.56
GLRX2	Amplification	47.62	2.21E-06	0.66
GMDS	Amplification	30.95	8.24E-12	1.54
GOLPH3L	Amplification	33.33	3.20E-08	0.94
GOLT1A	Amplification	38.1	8.98E-06	0.93

GORAB	Amplification	52.38	6.21E-07	1.02
GPR89A	Amplification	40.48	6.38E-14	1.12
HDGF	Amplification	40.48	3.28E-08	0.67
HEATR1	Amplification	35.71	1.97E-07	0.56
HEATR2	Amplification	33.33	3.71E-12	0.91
HHAT	Amplification	42.86	9.11E-07	0.78
HIBADH	Amplification	47.62	1.86E-07	0.54
HNRNPA2B1	Amplification	30.95	4.35E-07	0.51
HUS1	Amplification	42.86	6.12E-08	0.8
IARS2	Amplification	30.95	3.65E-09	0.79
ICA1	Amplification	42.86	2.56E-10	0.93
IGF2BP3	Amplification	30.95	3.23E-08	2.41
IGFBP3	Amplification	47.62	4.34E-06	1.52
IGSF9	Amplification	50	2.03E-11	1.56
IKBKE	Amplification	42.86	1.31E-06	0.52
INTS7	Amplification	38.1	1.60E-06	0.89
IQGAP3	Amplification	35.71	1.17E-07	1.55
IRF6	Amplification	47.62	3.34E-08	0.77
ISG20L2	Amplification	40.48	6.82E-06	0.61
JMJD4	Amplification	38.1	4.44E-08	0.61
JMJD8	Amplification	33.33	3.32E-06	0.51
KCNK1	Amplification	40.48	1.13E-06	1.31
KDM5B	Amplification	30.95	5.53E-09	1.07
KIF14	Amplification	30.95	5.16E-08	1.44
KLHL12	Amplification	30.95	1.06E-07	0.79
KLHL7	Amplification	40.48	1.78E-07	1.31
LAD1	Amplification	38.1	4.48E-08	1.44
LANCL2	Amplification	45.24	3.67E-07	0.98
LOC153684	Amplification	30.95	8.82E-07	0.96
LOC25845	Amplification	47.62	5.44E-07	0.91
LOC642852	Amplification	30.95	4.20E-06	1
LOC646762	Amplification	42.86	7.63E-07	0.75
LOC728613	Amplification	47.62	5.33E-06	0.63
LSM5	Amplification	30.95	4.14E-10	1.13
LYSMD1	Amplification	30.95	3.31E-06	0.67
MACC1	Amplification	50	1.57E-06	1.3
MAL2	Amplification	38.1	1.55E-06	0.94
MARCH6	Amplification	47.62	3.31E-07	0.73
METRN	Amplification	33.33	1.13E-06	0.7
METTL13	Amplification	47.62	3.87E-12	0.61
MIOS	Amplification	42.86	5.98E-07	0.67
MPZL1	Amplification	45.24	6.71E-08	0.76
MRPL24	Amplification	40.48	7.18E-09	0.81
MRPL36	Amplification	50	2.39E-09	0.8
MRPL55	Amplification	35.71	2.47E-09	0.74
MRPL9	Amplification	47.62	8.02E-11	1

MRPS14	Amplification	47.62	4.44E-08	0.66
MRPS24	Amplification	35.71	4.71E-14	0.99
MUT	Amplification	35.71	2.76E-06	0.68
NARFL	Amplification	30.95	8.96E-07	0.59
NEK2	Amplification	35.71	5.24E-10	2.38
NFE2L3	Amplification	30.95	6.20E-08	1.61
NIT1	Amplification	30.95	1.50E-06	0.48
NSL1	Amplification	33.33	1.16E-06	0.72
NSMCE2	Amplification	30.95	3.26E-07	0.64
NUPL2	Amplification	38.1	4.43E-12	1.15
OSBPL2	Amplification	30.95	5.87E-06	0.81
OSMR	Amplification	42.86	4.70E-06	1.21
PAIP1	Amplification	35.71	2.34E-09	1.21
PDCD6	Amplification	47.62	1.92E-06	0.74
PEX11B	Amplification	38.1	2.54E-07	0.79
PFDN2	Amplification	30.95	2.34E-06	0.55
PHF14	Amplification	42.86	2.27E-08	1.06
PIAS3	Amplification	40.48	6.94E-10	1.03
PIGM	Amplification	50	1.15E-06	0.7
PLEKHA6	Amplification	38.1	3.27E-08	1.35
PLEKHA8	Amplification	42.86	2.34E-11	1.84
POLD2	Amplification	40.48	7.34E-08	0.89
POLM	Amplification	38.1	1.49E-06	0.67
PRLR	Amplification	42.86	3.45E-06	1.7
PRUNE	Amplification	30.95	1.77E-07	0.65
PSMA2	Amplification	45.24	5.74E-06	0.67
PSMB4	Amplification	30.95	2.28E-08	0.66
PSMD4	Amplification	30.95	9.72E-07	0.51
PSMG3	Amplification	30.95	1.35E-12	1.39
PSPH	Amplification	35.71	8.56E-12	2.22
PVRL4	Amplification	30.95	5.57E-08	0.94
PYGO2	Amplification	33.33	1.93E-07	0.7
RAB40C	Amplification	35.71	2.44E-07	1.14
RABIF	Amplification	30.95	6.50E-11	0.68
RAD1	Amplification	40.48	3.98E-08	0.99
RAE1	Amplification	30.95	1.97E-13	0.97
RALGPS2	Amplification	42.86	2.70E-06	1.13
RBM8A	Amplification	38.1	1.34E-08	0.61
RHBDL1	Amplification	35.71	5.05E-07	0.76
RNASEN	Amplification	33.33	3.09E-07	0.74
RNF187	Amplification	33.33	9.60E-06	0.68
RPA3	Amplification	40.48	9.34E-07	1.01
RPS10P7	Amplification	33.33	3.94E-06	0.56
RPUSD1	Amplification	30.95	2.92E-06	0.4
S100A11	Amplification	57.14	1.12E-06	0.51
SAMD12	Amplification	35.71	2.33E-06	1.08

SCRN1	Amplification	42.86	9.48E-08	1.05
SCYL3	Amplification	50	7.44E-07	0.7
SDHA	Amplification	47.62	1.66E-07	0.51
SDHC	Amplification	33.33	3.85E-11	0.66
SEC61G	Amplification	54.76	2.55E-06	0.97
SETDB1	Amplification	35.71	6.85E-07	0.67
SFT2D2	Amplification	42.86	2.26E-06	0.66
SHC1	Amplification	33.33	2.86E-06	0.67
SIX1	Amplification	33.33	1.11E-08	3.06
SIX4	Amplification	33.33	2.50E-07	1.75
SLC37A1	Amplification	30.95	1.47E-07	0.56
SMG7	Amplification	45.24	5.22E-08	0.72
SMYD3	Amplification	33.33	6.95E-10	0.89
SNAP47	Amplification	38.1	2.69E-11	0.82
SNRPE	Amplification	30.95	4.21E-10	0.85
SPINK1	Amplification	30.95	3.17E-09	4.92
SRD5A1	Amplification	45.24	1.30E-12	2.28
STK31	Amplification	38.1	1.39E-08	1.4
STX16	Amplification	35.71	7.75E-06	0.87
STX6	Amplification	45.24	4.05E-06	0.7
SUB1	Amplification	35.71	5.65E-08	0.46
SUSD4	Amplification	35.71	9.14E-09	1.34
TAF2	Amplification	30.95	1.54E-06	0.6
TAF4	Amplification	30.95	1.63E-08	0.91
TAF5L	Amplification	30.95	4.48E-07	0.33
TARBP1	Amplification	40.48	1.37E-06	0.81
TARS	Amplification	38.1	2.03E-06	0.8
TAX1BP1	Amplification	47.62	6.34E-07	0.59
TBRG4	Amplification	40.48	1.50E-10	0.9
TDRKH	Amplification	50	5.33E-10	1.6
TFB2M	Amplification	30.95	9.00E-06	0.77
TH1L	Amplification	33.33	5.52E-11	1.25
TMCO1	Amplification	38.1	9.33E-07	0.77
TMED4	Amplification	30.95	6.49E-07	0.62
TMEM106B	Amplification	50	4.95E-10	1.89
TMEM184A	Amplification	30.95	1.73E-07	0.8
TMEM9	Amplification	40.48	9.56E-09	0.94
TOR3A	Amplification	33.33	3.28E-08	0.72
TRAPPC9	Amplification	30.95	2.59E-06	0.63
TRIM11	Amplification	35.71	7.46E-07	0.54
TRMT5	Amplification	33.33	1.27E-08	0.94
TTC13	Amplification	30.95	2.32E-07	0.9
UBE2D4	Amplification	35.71	3.29E-07	0.56
UBE2Q1	Amplification	35.71	1.88E-08	0.46
UBE2T	Amplification	40.48	1.13E-12	1.99
UFC1	Amplification	30.95	9.82E-07	0.51

VAPB	Amplification	33.33	9.43E-08	0.89
VPS45	Amplification	38.1	1.05E-10	0.9
VPS72	Amplification	30.95	2.42E-10	0.8
WDR24	Amplification	33.33	3.90E-07	0.72
XPR1	Amplification	47.62	7.10E-11	1.63
YKT6	Amplification	40.48	7.16E-10	0.81
YTHDF1	Amplification	40.48	5.17E-08	1.12
ZC3H11A	Amplification	30.95	2.75E-06	0.53
ZNF238	Amplification	42.86	5.55E-06	0.96
ZNF275	Amplification	30.95	8.08E-06	0.66
ZNF281	Amplification	30.95	4.06E-08	0.94
ZNF572	Amplification	33.33	1.46E-06	0.52
ZNF672	Amplification	38.1	1.94E-06	0.54
ZNF692	Amplification	38.1	3.05E-06	0.86
ABHD6	Deletion	30.95	2.70E-10	-1.01
ACACB	Deletion	30.95	1.71E-06	-1.43
ADIPOR2	Deletion	30.95	3.62E-08	-0.92
AIF1L	Deletion	45.24	2.42E-08	-1.44
AKAP12	Deletion	35.71	9.48E-10	-2.39
AKNA	Deletion	30.95	1.72E-07	-0.92
AMD1	Deletion	42.86	3.66E-06	-0.71
ANKDD1A	Deletion	30.95	2.47E-07	-0.94
ANKRD11	Deletion	30.95	2.20E-06	-0.48
ANKRD29	Deletion	30.95	1.02E-15	-3.4
ANP32B	Deletion	30.95	1.56E-06	-0.57
ARHGAP19	Deletion	30.95	1.58E-06	-0.46
ARHGEF10	Deletion	30.95	1.01E-13	-1.44
ARHGEF15	Deletion	50	9.16E-11	-1.34
ARHGEF3	Deletion	30.95	1.62E-08	-0.99
ARL3	Deletion	38.1	8.44E-07	-0.44
ASAH1	Deletion	33.33	9.01E-06	-0.61
ASPA	Deletion	38.1	4.73E-11	-1.89
AVPI1	Deletion	30.95	3.35E-07	-0.86
BCL6B	Deletion	45.24	2.63E-09	-1.14
C10orf67	Deletion	30.95	7.94E-09	-1.02
C13orf1	Deletion	38.1	1.39E-11	-1.58
C17orf91	Deletion	52.38	4.18E-08	-1.66
C18orf8	Deletion	33.33	6.81E-06	-0.63
C19orf59	Deletion	38.1	2.19E-09	-2.92
C3orf42	Deletion	30.95	1.92E-06	-0.46
C3orf63	Deletion	35.71	3.97E-06	-0.86
C5AR1	Deletion	45.24	3.09E-06	-1.68
C8orf4	Deletion	33.33	1.52E-06	-1.31
CALHM2	Deletion	30.95	2.48E-07	-0.85
CAMP	Deletion	47.62	3.21E-06	-0.85
CARD8	Deletion	38.1	6.23E-08	-1.32

CCBE1	Deletion	38.1	1.08E-15	-2.1
CCDC25	Deletion	40.48	6.90E-07	-0.7
CCDC68	Deletion	35.71	5.43E-09	-1.69
CDK9	Deletion	42.86	2.32E-06	-0.33
CDKN2B	Deletion	38.1	4.96E-13	-2.52
CFD	Deletion	30.95	2.99E-11	-2.67
CHMP1B	Deletion	33.33	5.38E-08	-1.11
CLIC3	Deletion	30.95	2.52E-11	-2.19
CLU	Deletion	42.86	4.38E-09	-2.17
CNN1	Deletion	40.48	4.74E-06	-1.08
COX7A1	Deletion	30.95	1.67E-09	-1.46
CSGALNACT1	Deletion	30.95	3.42E-06	-1.59
CYB5R3	Deletion	30.95	9.99E-06	-0.55
DAB2IP	Deletion	30.95	8.69E-10	-0.97
DCC	Deletion	30.95	1.75E-06	-1.09
DDX19B	Deletion	35.71	4.43E-06	-0.72
DENND2A	Deletion	30.95	6.93E-11	-1.56
DENND4C	Deletion	33.33	4.28E-07	-0.71
DHDPSL	Deletion	33.33	3.22E-06	-0.63
DLC1	Deletion	30.95	3.58E-11	-2.44
DNAH12	Deletion	30.95	2.51E-06	-1.7
DNAJB12	Deletion	35.71	3.00E-06	-0.37
DNASE1L3	Deletion	30.95	2.09E-09	-2.08
DOCK6	Deletion	38.1	3.14E-06	-0.79
DPYSL2	Deletion	42.86	2.72E-08	-1.36
EEF1A1	Deletion	35.71	8.26E-07	-1.06
EFHA2	Deletion	33.33	3.24E-09	-1.87
EHD2	Deletion	45.24	6.20E-06	-0.85
ELAVL1	Deletion	38.1	2.17E-06	-0.54
EMR1	Deletion	40.48	1.52E-06	-1.23
ENG	Deletion	42.86	9.38E-08	-1.04
EPB49	Deletion	38.1	1.72E-06	-0.54
ERICH1	Deletion	38.1	2.56E-07	-0.9
EVI5	Deletion	30.95	5.08E-09	-0.86
FAM101B	Deletion	45.24	4.49E-07	-1.14
FAM116A	Deletion	40.48	3.38E-09	-0.79
FAM129B	Deletion	42.86	2.80E-06	-0.58
FAM167A	Deletion	30.95	5.40E-07	-1.38
FAM189A2	Deletion	30.95	1.33E-12	-2.1
FAM82A2	Deletion	40.48	1.83E-06	-0.5
FBP1	Deletion	40.48	7.04E-06	-0.96
FGD3	Deletion	33.33	1.51E-08	-1.19
FNTA	Deletion	38.1	2.38E-06	-0.51
FOSB	Deletion	45.24	1.19E-07	-2.91
FOXO1	Deletion	30.95	1.74E-08	-1.3
FRMD4B	Deletion	30.95	2.17E-06	-0.98

FUT1	Deletion	45.24	8.08E-08	-1.06
FXR2	Deletion	50	1.70E-06	-0.41
GABARAPL1	Deletion	33.33	1.34E-08	-1.17
GADD45B	Deletion	40.48	3.50E-07	-1.45
GAS7	Deletion	40.48	1.51E-06	-0.98
GATA6	Deletion	40.48	2.50E-08	-2.39
GFRA2	Deletion	40.48	2.33E-08	-0.71
GLIPR2	Deletion	30.95	5.16E-12	-1.43
GNA11	Deletion	40.48	7.99E-06	-0.42
GOLGA7	Deletion	38.1	6.14E-10	-0.76
GPRC5A	Deletion	35.71	2.02E-08	-1.72
HIF3A	Deletion	47.62	9.15E-08	-1.31
HMGB1	Deletion	33.33	1.28E-13	-1.2
IER2	Deletion	38.1	1.70E-06	-0.98
IFT88	Deletion	30.95	1.30E-08	-0.73
IL17D	Deletion	30.95	6.65E-09	-1.22
INO80C	Deletion	30.95	4.05E-07	-1.05
INPP5K	Deletion	52.38	1.42E-09	-0.74
JUND	Deletion	30.95	2.65E-09	-0.81
KANK2	Deletion	38.1	3.19E-08	-1.22
KANK3	Deletion	38.1	8.57E-15	-2.14
KATNA1	Deletion	33.33	1.67E-07	-0.67
KIAA0427	Deletion	38.1	8.62E-12	-0.88
KIF13B	Deletion	40.48	4.99E-07	-0.69
KLF2	Deletion	30.95	3.15E-09	-1.81
KLF4	Deletion	54.76	2.29E-07	-2.45
KLHL9	Deletion	33.33	9.56E-07	-0.83
KLK10	Deletion	35.71	2.44E-08	-1.7
KPNA3	Deletion	38.1	1.29E-06	-0.89
LAMC3	Deletion	42.86	3.36E-07	-0.84
LATS2	Deletion	47.62	5.80E-09	-1.42
LDLR	Deletion	38.1	4.47E-06	-1.66
LGI3	Deletion	38.1	1.52E-10	-1.42
LIMD1	Deletion	30.95	3.04E-07	-0.92
LPL	Deletion	35.71	1.29E-07	-1.9
LRRFIP2	Deletion	33.33	7.26E-07	-0.5
LTBP4	Deletion	30.95	6.77E-11	-1.81
MAP3K8	Deletion	30.95	2.31E-07	-1.92
MAP4	Deletion	47.62	1.55E-06	-0.65
MAPK1	Deletion	30.95	1.26E-07	-0.57
MAPKAP1	Deletion	30.95	9.16E-06	-0.52
MAPRE2	Deletion	30.95	2.87E-08	-0.89
MPHOSPH8	Deletion	35.71	1.67E-07	-0.79
MPRIP	Deletion	33.33	4.87E-06	-0.59
MTUS1	Deletion	35.71	1.40E-06	-0.83
MYADM	Deletion	38.1	7.41E-11	-1.65

MYH10	Deletion	35.71	1.26E-10	-1.58
MYO1C	Deletion	54.76	1.33E-06	-0.86
MYO5B	Deletion	30.95	1.18E-07	-1.25
MYST4	Deletion	30.95	8.65E-07	-0.86
NDEL1	Deletion	35.71	1.06E-08	-0.85
NEDD4L	Deletion	35.71	1.47E-10	-2.52
NEK3	Deletion	35.71	4.54E-07	-0.89
NFATC1	Deletion	35.71	2.94E-06	-0.99
NPDC1	Deletion	30.95	3.54E-07	-0.97
NUP214	Deletion	45.24	5.42E-06	-0.4
OGG1	Deletion	38.1	8.07E-06	-0.59
OLFM1	Deletion	30.95	2.95E-06	-1.24
OLFML2A	Deletion	38.1	1.00E-08	-1.41
OSBPL1A	Deletion	30.95	2.42E-06	-0.98
OTUD1	Deletion	33.33	6.53E-11	-1.44
PAFAH1B1	Deletion	54.76	1.10E-06	-0.74
PALM2-AKAP2	Deletion	30.95	2.08E-10	-2.48
PCM1	Deletion	33.33	4.89E-10	-1.77
PCMT1	Deletion	33.33	8.26E-06	-0.65
PDLIM2	Deletion	42.86	1.63E-11	-1.32
PEBP4	Deletion	42.86	5.42E-12	-3.15
PIK3R5	Deletion	38.1	6.55E-06	-0.84
PIP5K1B	Deletion	30.95	1.37E-06	-2.19
PLAC2	Deletion	40.48	4.24E-10	-1.42
PLAC9	Deletion	35.71	2.03E-11	-2.43
PLEKHA2	Deletion	35.71	1.54E-07	-0.82
PMP22	Deletion	30.95	9.92E-11	-1.41
POLDIP3	Deletion	30.95	5.80E-08	-0.59
PPP1R14A	Deletion	30.95	7.18E-13	-2.31
PPP1R15A	Deletion	42.86	1.83E-11	-1.89
PPP2CB	Deletion	50	5.01E-07	-1.06
PPP3CC	Deletion	40.48	1.55E-07	-0.88
PPP6C	Deletion	52.38	2.65E-08	-0.94
PQLC1	Deletion	38.1	6.08E-08	-0.71
PROSC	Deletion	38.1	5.87E-07	-0.92
PRX	Deletion	30.95	3.56E-07	-0.99
PSIP1	Deletion	47.62	2.50E-08	-1.16
PTGDS	Deletion	30.95	3.06E-11	-2.36
R3HCC1	Deletion	42.86	8.01E-06	-0.55
RAB11FIP1	Deletion	45.24	1.65E-10	-1.78
RAPGEF1	Deletion	30.95	3.72E-09	-0.99
RASIP1	Deletion	45.24	1.74E-14	-2.12
RBPMS	Deletion	42.86	8.63E-06	-0.79
RFX2	Deletion	33.33	2.60E-06	-1.3
RICH2	Deletion	35.71	5.95E-12	-1.2
RILPL2	Deletion	35.71	4.99E-06	-0.69

RNF125	Deletion	54.76	9.15E-08	-1.32
RNF38	Deletion	45.24	9.75E-10	-0.73
RRAGA	Deletion	30.95	4.28E-06	-0.64
RRAS	Deletion	38.1	3.35E-10	-1.33
SAP18	Deletion	45.24	2.31E-10	-1.06
SASH1	Deletion	30.95	5.76E-09	-1.89
SAT2	Deletion	50	2.53E-07	-0.67
SCAI	Deletion	45.24	2.14E-08	-1.77
SCARA3	Deletion	40.48	5.94E-08	-1.46
SCARA5	Deletion	45.24	2.19E-09	-2.8
SEC63	Deletion	30.95	6.55E-06	-1.15
SEMA3G	Deletion	30.95	4.17E-06	-1.81
SETX	Deletion	35.71	3.26E-06	-0.44
SFTPC	Deletion	38.1	3.21E-10	-3.92
SFTPD	Deletion	33.33	3.13E-08	-3.22
SH2D3C	Deletion	42.86	1.45E-12	-1.83
SH3BP5	Deletion	30.95	1.52E-10	-1.16
SH3GL2	Deletion	30.95	5.96E-07	-1.29
SLC19A3	Deletion	30.95	5.01E-07	-1.27
SLC25A25	Deletion	45.24	5.16E-08	-1.06
SLC44A2	Deletion	38.1	9.15E-07	-0.8
SLC6A16	Deletion	40.48	3.16E-07	-1.05
SLMAP	Deletion	40.48	2.50E-08	-0.99
SMAD7	Deletion	45.24	3.17E-06	-1.08
SOX7	Deletion	30.95	4.60E-10	-3.26
SPNS2	Deletion	35.71	5.61E-07	-0.86
SPOCK2	Deletion	30.95	3.47E-19	-3.01
SRGN	Deletion	30.95	7.94E-06	-0.72
ST6GALNAC6	Deletion	42.86	1.08E-09	-0.92
TACC1	Deletion	33.33	1.32E-13	-1.44
TACC2	Deletion	33.33	3.43E-07	-1.2
TBXA2R	Deletion	42.86	5.61E-06	-0.73
TCF4	Deletion	30.95	3.33E-11	-1.53
TEK	Deletion	30.95	2.72E-10	-2.67
TEKT1	Deletion	42.86	8.37E-06	-1.23
TFRC	Deletion	30.95	1.02E-06	-1.38
TGFBR3	Deletion	30.95	2.03E-15	-2.78
TJP2	Deletion	33.33	5.49E-11	-1.42
TMEM146	Deletion	40.48	1.46E-06	-1.84
TMEM88	Deletion	40.48	2.06E-10	-1.17
TNFRSF1A	Deletion	35.71	2.02E-06	-0.58
TNFSF12	Deletion	50	6.41E-06	-0.59
TNNC1	Deletion	30.95	2.38E-15	-3.6
TOM1L2	Deletion	40.48	1.55E-10	-1.03
TRAK1	Deletion	30.95	2.04E-06	-0.59
TRIM35	Deletion	40.48	8.63E-06	-0.51

TRPV2	Deletion	42.86	1.09E-07	-1.05
ULK2	Deletion	35.71	7.49E-06	-0.88
USHBP1	Deletion	30.95	9.60E-06	-0.42
USP8	Deletion	33.33	2.30E-07	-0.72
VPS4B	Deletion	35.71	6.65E-06	-0.6
VWF	Deletion	33.33	4.38E-13	-2.27
WAPAL	Deletion	45.24	4.29E-07	-0.57
WDR47	Deletion	33.33	2.52E-06	-0.65
XPO4	Deletion	30.95	1.14E-07	-0.61
ZBTB34	Deletion	40.48	1.75E-08	-0.86
ZBTB4	Deletion	45.24	1.68E-08	-0.91
ZFP36	Deletion	30.95	2.34E-07	-1.52
ZNF331	Deletion	30.95	3.23E-08	-1.87
ZNF596	Deletion	35.71	4.80E-06	-0.55
ZRANB1	Deletion	35.71	8.50E-08	-0.6
ZZEF1	Deletion	40.48	5.21E-06	-0.77
