# Supplementary material for: Integrated Analyses of Copy Number Variations and Gene Expression in Lung Adenocarcinoma
Source: PLoS One. 2011 Sep 14;6(9):e24829. doi: 10.1371/journal.pone.0024829 (PMC3173487; doi:10.1371/journal.pone.0024829)
Supplement: Table S2 — Empirical p -values of the canonical pathways in GSE4573a (PDF) [file pone.0024829.s007.pdf]

Table S2. Empirical *p*-values of the canonical pathways in GSE4573[a]

Canonical Pathway	GSE4573
**IL-3 Signaling**	0.1522
**Aminoacyl-tRNA Biosynthesis**	0.3499
**EIF2 Signaling**	0.2816
**PTEN Signaling**	0.0311
**Renal Cell Carcinoma Signaling**	0.1679
**Oncostatin M Signaling**	0.0188
**Ephrin Receptor Signaling**	0.0124

[a]The significance levels were determined by comparison with null baselines created by random selections.
