# Supplementary material for: Integrated Analyses of Copy Number Variations and Gene Expression in Lung Adenocarcinoma
Source: PLoS One. 2011 Sep 14;6(9):e24829. doi: 10.1371/journal.pone.0024829 (PMC3173487; doi:10.1371/journal.pone.0024829)
Supplement: Table S3 — Sample characteristics of lung cancer patients examined by both Affymetrix SNP6.0 and Affymetrix U133plus 2.0 arrays. (PDF) [file pone.0024829.s008.pdf]

**Table S3. Sample characteristics of lung cancer patients examined by both Affymetrix SNP6.0 and Affymetrix U133plus 2.0 arrays.**

| Characteristics | Microarray |
|---|---|
| **Sample Number** | 42 |
| **Age** | 62.0±10 |
| **Histology** | |
| **Adenocarcinoma** | 42 (100%) |
| **Gender** | |
| **Female** | 42 (100%) |
| **Stage** | |
| **IA** | 7 (17%) |
| **IB** | 14 (33%) |
| **IIA** | 3 (7%) |
| **IIB** | 6 (14%) |
| **IIIA** | 7 (17%) |
| **IIIB** | 4 (10%) |
| **IV** | 1 (2%) |
