# Supplementary material for: Integrated Analyses of Copy Number Variations and Gene Expression in Lung Adenocarcinoma
Source: PLoS One. 2011 Sep 14;6(9):e24829. doi: 10.1371/journal.pone.0024829 (PMC3173487; doi:10.1371/journal.pone.0024829)
Supplement: Table S4 — Genes involved in the IL-3 signaling and ephrin receptor signaling pathways. (PDF) [file pone.0024829.s009.pdf]

**Table S4. Genes involved in the IL-3 signaling and ephrin receptor signaling Pathways.**

| Pathway | Number | Symbol |
|---|---|---|
| **IL-3 Signaling** | 7 | *SHC1, FOXO1, MAPK1, RRAS, PI3KR5, PPPCC, RAPGEF1* |
| **Ephrin Receptor Signaling** | 10 | *SHC1,GNA11,MAPK1,RRAS,ARHGEF15, EFNA3,EFNA4, EFNA5, SH2D3C,RAPGEF1* |
